# Human Dental Pulp Tissue during Orthodontic Tooth Movement: An Immunofluorescence Study

**DOI:** 10.3390/jfmk5030065

**Published:** 2020-08-22

**Authors:** Giovanna Vermiglio, Antonio Centofanti, Giovanni Matarese, Angela Militi, Marco Matarese, Alba Arco, Fabiana Nicita, Giuseppina Cutroneo

**Affiliations:** 1 Department of Biomedical and Dental Sciences and Morphofunctional Imaging, University of Messina, 98124 Messina, Italy; centofantia@unime.it (A.C.); gmatarese@unime.it (G.M.); amiliti@unime.it (A.M.); mataresem@gmail.com (M.M.); aarco@unime.it (A.A.); fabin92@hotmail.it (F.N.); 2 Department of Clinic and Experimental Medicine, University of Messina, 98124 Messina, Italy; gcutroneo@unime.it

**Keywords:** human dental pulp, orthodontic tooth movement, immunofluorescence, extracellular matrix proteins

## Abstract

The orthodontic tooth movement is the last step of several biological processes that take place after the application of external forces. During this process, dental pulp tissue is subjected to structural and protein expression modifications in order to maintain their integrity and functional morphology. The purpose of the present work was to perform an in vivo study, evaluating protein expression modifications in the human dental pulp of patients that have undergone orthodontic tooth movement due to pre-calibrated light force application for 30 days. Dental pulp samples were extracted from molars and premolars of the control group and after 7 and 30 days of treatment; the samples were then processed for immunofluorescence reactions using antibodies against fibronectin, collagen I and vascular endothelial growth factor (VEGF). Our results show that, after 7 days of treatment, all tested proteins change their pattern expression and will reset after 30 days. These data demonstrate that the dental pulp does not involve any irreversible iatrogenic alterations, supporting the efficacy and safety of using pre-calibrated force application to induce orthodontic tooth movement in clinical practice.

## 1. Introduction

The orthodontic tooth movement (OTM) could be defined as the results of tooth biological system response to the application of an externally force; all the biological responses that take place after force application lead to bone remodeling that is necessary for OTM [1,2].

The size of the biological response depends on the application time, force magnitude and force distribution [3]; in fact, different force distribution patterns could determine different type of tissue reactions. By that, several studies focused on evaluating tissue reaction to force appliance [4,5] and iatrogenic sequelae to orthodontic force have been detected.

The prolonged force appliance could determine dental pulp alterations that may culminate in a loss of vitality due to pulpal blood flow alterations [6,7,8,9]. The characteristics of applied orthodontic forces, such as magnitude, appliance time and distribution, could contribute to blood flow disturbance [7,9,10,11] and make the alteration reversibly or irreversibly.

The literature shows conflicting results about the correlations of pulp changes incident to orthodontic force. Some reports suggested permanent damage to pulpal tissue from orthodontic force as tissue calcification and vascular alteration with vascular stasis and pulp necrosis [12] but others supported no significant long-lasting effects of dental pulp [13,14]. The clinical impact of these studies was to determine whether any alterations in pulpal tissues could affect the long-term vitality of the teeth.

Despite literature showing various results about pulp reaction to orthodontic tooth movement, it is unanimously accepted that the size of dental pulp modification or injury is directly proportional to the applied forces that in adults has to be between 50 and 100 cN [7,15,16].

Here, we performed an in vivo study on human pulp tissue proteins as vascular endothelial growth factor (VEGF), collagen I and fibronectin over a period of 30 days by the application of pre-calibrated light orthodontic force on the maxillary and mandibular premolars.

## 2. Materials and Methods

### 2.1. Patients Selection

For this study, we analyzed the in vivo human samples of dental pulp of 72 first maxillary and mandibular premolars of 18 subjects, scheduled for orthodontic treatment, at the Department of Dentistry of Messina University, Italy. The study was carried out with the informed consent of patients (or their parents, in case of underage patients), and the experimental procedures described here were authorized by Ethical Committee number XX 35-17 22/05/2017. All patients met the following criteria: (1) all 4 first premolars had to be extracted for orthodontic reasons; (2) general health good, and the periodontium was healthy with no radiographic evidence of bone loss, no gingival inflammation; (3) no antibiotics or anti-inflammatory drugs were used in the month before the study.

All subjects received repeated oral hygiene instruction about how to use a toothbrush, dental floss and interdental brush before the placement of the orthodontic appliance. The subjects, 11 females and 7 males (mean age 15,4 years; range, 13–18 years), not matched for age and sex, were randomly divided into 3 main groups, each of which consisted of 3 subjects. Group I: the premolars, not subjected to orthodontic force, were extracted and dental pulp sampling was used as a control; group II: the premolars were extracted 7 days after the application of orthodontic force and the dental pulp sampling was analyzed; group III: the premolars were extracted 30 days after the application of orthodontic force and the dental pulp was analyzed.

### 2.2. Clinical Procedures

For the application of orthodontic force during experimental tooth movement, we directly bonded 0.022 inc, LP tubes and Master brackets (American Orthodontics Sheboygan, WI, USA) to the buccal surface of the maxillary first premolars and molars. The premolars were subjected to a buccal directed tipping force (50 g) with a Nichel Titanium closed coil spring (American Orthodontics). Bonding of the brackets, bending of the springs, and calibration of force with a strain gauge (Dentaurum, Ispringen, Germany) was performed by the same clinician. After the experimental period, the premolars were extracted by the same surgeon with no surgical trauma to the root. The dental pulp samples have been obtained opening the dental cavity by a rose-head-bur and extracting the pulp tissue with a barbed-broaches.

### 2.3. Immunofluorescence

The dental pulp samples were fixed in 3% paraformaldehyde in a 0.2 M phosphate buffer at pH 7.4. After numerous rinses in 0.2 M phosphate buffer and saline phosphate buffer (BPS), they were infiltrated with 12% and 18% sucrose and frozen in liquid nitrogen. By cryostat, the samples were cut into 10-µm-thick sections collected on glass slides coated with 0.5% gelatin and 0.005% chromium potassium sulphate. On these sections, four different single localization reactions were performed to mark the Collagen I, Fibronectin and VEGF as our standard protocol [17]. The following primary antibodies were used: mouse monoclonal anti-collagen I antibody, diluted 1:400 (Sigma Aldrich, S. Louis, MS, USA) mouse monoclonal anti fibronectin, diluted 1:200 (Sigma Aldrich) and anti-VEGF antibody diluted 1:400 (Novus Biologicals, Littleton, CO, USA). All primary antibodies were demonstrated with Texas Red conjugated with IgG anti-mouse, diluted 1:100 (Jackosn ImmunoResearch Laboratories, West Grove, PA, USA); the fluorochrome was applied for 1 h at room temperature. Then sections were observed and photographed using a ZEISS LSM 5 DUO confocal microscope (Carl-Zeiss, Jena, Germany) [18,19]. For the detection of images Laser Argon (458 and 488 nm) was utilized. All images were digitized at resolution of 8 bits into an array of 2048 × 2048 pixels. Optical sections of fluorescence specimens were obtained using a HeNe laser (543 nm) and an Argon Laser (458) at a 1 min 2 sec scanning speed with up to 8. Thick sections (1.50 µm) were obtained using a pinhole of 250. Contrast and brightness were established by examination.

### 2.4. Statistic Analysis

Zeiss LSM 5 DUO confocal microscope observations were analyzed by an internal software for image analysis, included in the CLSM software and named “Histo”, measuring the distribution of pixel intensity of all areas corresponding to each section; pixel intensities were converted in a data table indicating values of single pixel intensity. By this software, we analyzed 180 optical fields for each protein (10 optical field for patient) using for all observation the same confocal parameters, and a mean and standard deviation for each protein expression were obtained. The mean of each protein expression, at 7, 14 and 30 days, respectively, was used to create graphics. We used the ANOVA test (Analysis of Variance) to compare the means obtained at 7, 14 and 30 days for each protein and the *p*-value was calculated; the null hypothesis is that there is no difference among the means obtained at 7, 14 and 30 days of treatment, for each tested proteins.

## 3. Results and Discussions

The orthodontic tooth movement requires the application of force on the teeth and it generates mechanical stress in several structures as pulp, dentine, bone and periodontal ligament, which involves in several types of biological responses [20].

In the field of orthodontics, it is very important to avoid irreversible modification that could be induced by force application. By that, in recent years, the alterations in pulp vasculature and blood flow in response to orthodontic force have obtained much attention.

Several studies have been focused on morphological and protein changes in the bone and periodontal ligament during orthodontic tooth movement [2,21,22,23,24,25,26]; recently, the literature has also been paying attention to dental pulp response to low force application.

The aim of the present study was to evaluate the protein expression aspects of dental pulp from teeth undergone to light forces appliance. The pulp was extracted at 7 and 30 days of treatment. In particular, we examined the expression of extracellular matrix protein, as collagen I and fibronectin, to have information about reparative and regenerative processes and we also examined the vascular endothelial growth factor to analyze if angiogenic events take place.

**Figure 1:**
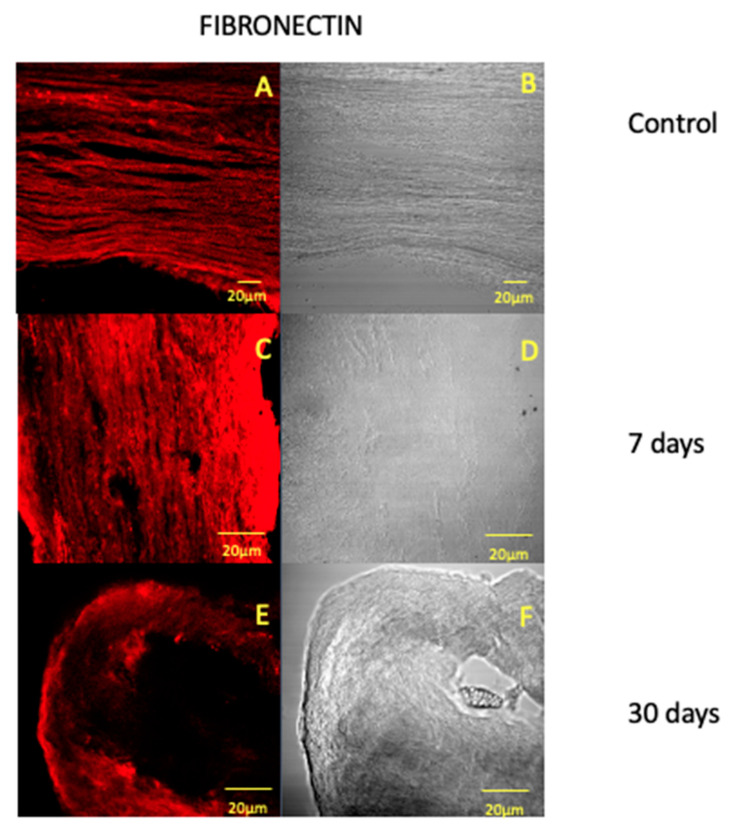
Compound panel of immunofluorescence reaction pictures that show fibronectin staining pattern in human dental pulp after 7 (**A**), 14 (**C**) and 30 (**E**) days after pre-calibrated force application and the corresponding transmitted light observations (**B**,**D**,**F**). The following magnifications have been used: 40× (**A**,**B**), 20× (**C**–**F**).

**Figure 2:**
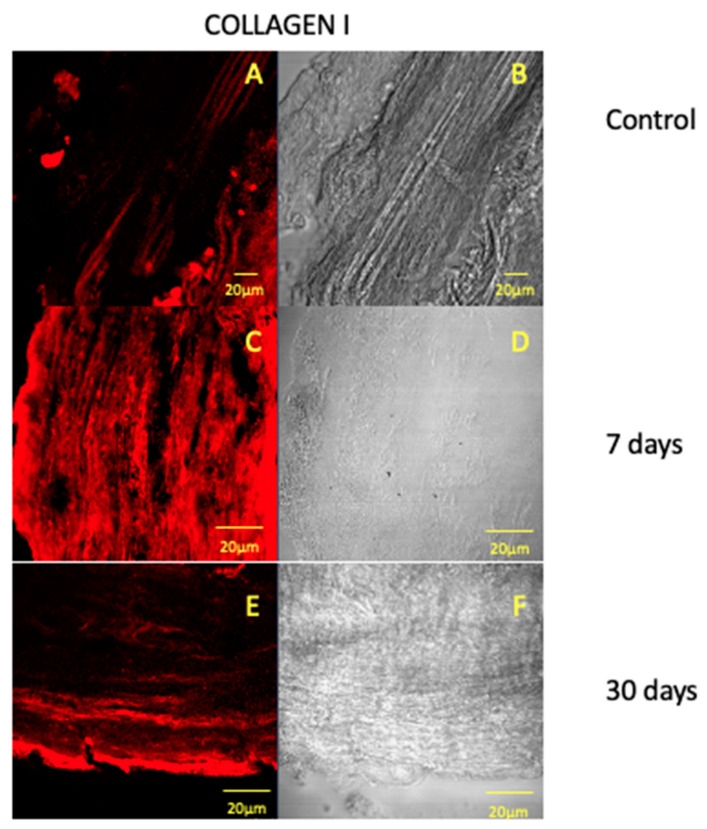
Compound panel of immunofluorescence reaction pictures that show collagen I staining pattern (red channel) in human dental pulp after 7 (**A**), 14 (**C**) and 30 (**E**) days after pre-calibrated force application and the corresponding transmitted light observations (**B**,**D**,**F**). The following magnifications have been used: 40× (**A**,**B**), 20× (**C**–**F**).

**Figure 3:**
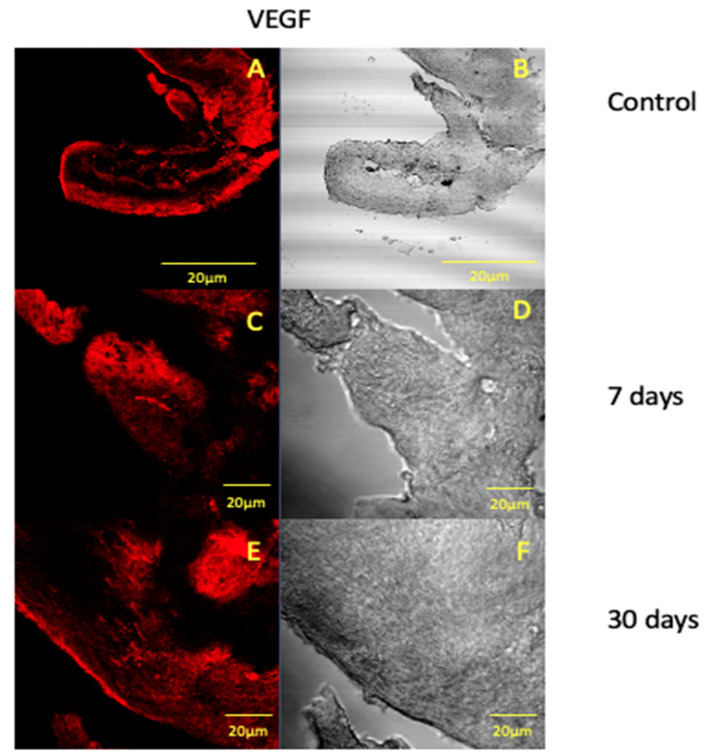
Compound panel of immunofluorescence reaction pictures that show VEGF staining pattern in human dental pulp after 7 (**A**), 14 (**C**) and 30 (**E**) days after pre-calibrated force application and the corresponding transmitted light observations (**B**,**D**,**F**). The following magnifications have been used: 10× (**A**,**B**), 20× (**C**–**F**).

The single localization immunofluorescence results show the presence of collagen I (Figure 1A), fibronectin (Figure 2A) and VEGF (Figure 3A) staining pattern in the control pulp samples as evidenced by a clearly detectable fluorescence signal of all proteins along the pulp tissue. The fluorescence pattern for fibronectin shows to increase after 7 days of treatment (Figure 1C); after 30 days, the pattern returns to be similar to the control (Figure 1E). It is well known that fibronectin is an important extracellular matrix constituent and it is highly produced from fibroblasts during reparative processes; the elevate fibronectin production from fibroblasts after mechanical stimuli application could be justified by the known elevate intrinsic repair capacity of dental pulp [27,28]. These data also suggest that reparative processes take place mainly in the first 7 days of force application; that is also in accordance with previous results that have shown an increased expression of fibronectin in the periodontal ligament during the initial phase of OTM [2].

Our results have also shown that the fluorescence pattern for collagen I increases after 7 days of treatment (Figure 2C) and the signal returns to be similar to the control after 30 days of treatment (Figure 2E). Collagen I is a very important structural protein of the extracellular matrix and its increased expression in the initial phase of force application suggests that collagen synthesis processes take place in response to force-induced tissue remodeling. After 30 days, the fluorescence pattern for collagen I is similar to the control and this data suggest that, after this period of force application, the synthesis of collagen I is restored. Both collagen I and fibronectin results are in accordance with literature that describe an association between an increased extracellular matrix proteins synthesis and orthodontic forces application [29,30].

We have observed that the force application determines the reduction in the VEGF fluorescence pattern after 7 days (Figure 3C), and it increases after 30 days of treatment (Figure 3E), suggesting that the dental pulp highly produces this factor in the late phase of treatment. These data show that the mechanical force does not induce irreversible blood flow alterations in dental pulp but it induces the VEGF production and an increase in the number of blood vessels. Our results also show that VEGF production is as time dependent as collagen I and fibronectin and that the vascular regenerative processes use to take place in the late phase of OTM, as we have already demonstrated in other dental tissues that have undergone force application [1,2,21,31,32,33,34]. The present work results show that reparative processes take place in human dental pulp that has undergone mechanical forces for 30 days. All the immunofluorescence data were supported by statistical analyses that have shown a significative statistical difference among the means of expression pattern obtained at 7, 14 and 30 days of treatment, for each tested protein, with the *p*-value < 0.05, and the null hypothesis was rejected.

**Figure 4:**
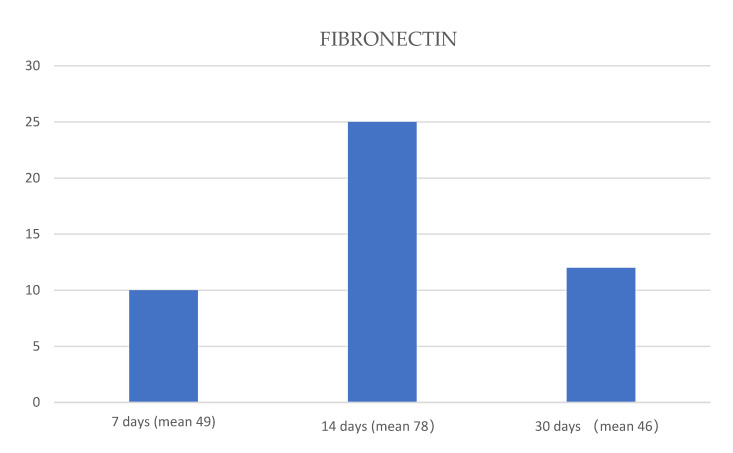
Graphic that shows the means of fibronectin expression (blue columns) at 7, 14 and 30 days. The Analysis of Variance (ANOVA) test showed a significant statistical difference among the means obtained at 7, 14 and 30 days of treatment (*p*-value < 0.05).

**Figure 5:**
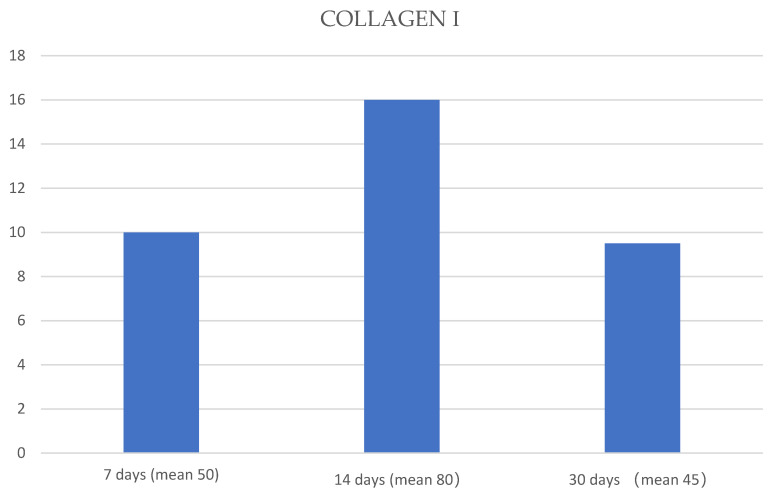
Graphic that shows the means of collagen I expression (blue columns) at 7, 14 and 30 days. The ANOVA test showed a significant statistical difference among the means obtained at 7, 14 and 30 days of treatment (*p*-value < 0.05).

**Figure 6:**
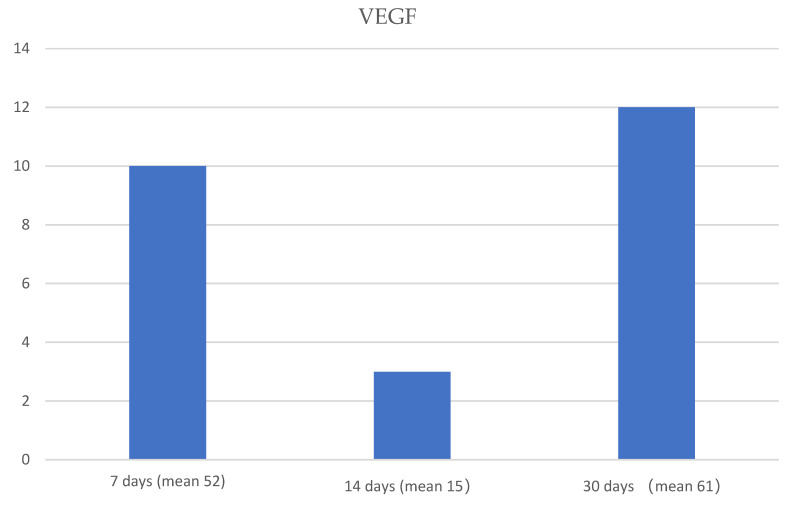
Graphic that shows the means of VEGF expression (blue columns) at 7, 14 and 30 days. The ANOVA test showed a significant statistical difference among the means obtained at 7, 14 and 30 days of treatment (*p*-value < 0.05).

The mean of each protein expression, at 7, 14 and 30 days, respectively, was used to create graphics (Figure 4, Figure 5 and Figure 6).

## 4. Conclusions

The present in vivo study, performed on human dental pulp during OTM, shows that, after 7 days of force application, the expression pattern of VEGF, fibronectin and collagen I changes, and it will reset after 30 days of treatment. This demonstrates that the dental pulp does not involve any irreversible iatrogenic alterations, supporting the efficacy and the safety of using pre-calibrated force application to induce orthodontic tooth movement.
